# Annotation-free prediction of microbial dioxygen utilization

**DOI:** 10.1128/msystems.00763-24

**Published:** 2024-09-04

**Authors:** Avi I. Flamholz, Joshua E. Goldford, Philippa A. Richter, Elin M. Larsson, Adrian Jinich, Woodward W. Fischer, Dianne K. Newman

**Affiliations:** 1 Division of Biology and Biological Engineering, California Institute of Technology, Pasadena, California, USA; 2 Division of Geological & Planetary Sciences, California Institute of Technology, Pasadena, California, USA; 3 Skaggs School of Pharmacy and Pharmaceutical Sciences, University of California San Diego, San Diego, California, USA; 4 Department of Chemistry and Biochemistry, University of California at San Diego, San Diego, California, USA; Monash University, Melbourne, Victoria, Australia

**Keywords:** oxygen, physiology, biogeochemistry, genome analysis, machine learning

## Abstract

Aerobes require dioxygen (O_2_) to grow; anaerobes do not. However, nearly all microbes—aerobes, anaerobes, and facultative organisms alike—express enzymes whose substrates include O_2_, if only for detoxification. This presents a challenge when trying to assess which organisms are aerobic from genomic data alone. This challenge can be overcome by noting that O_2_ utilization has wide-ranging effects on microbes: aerobes typically have larger genomes encoding distinctive O_2_-utilizing enzymes, for example. These effects permit high-quality prediction of O_2_ utilization from annotated genome sequences, with several models displaying ≈80% accuracy on a ternary classification task for which blind guessing is only 33% accurate. Since genome annotation is compute-intensive and relies on many assumptions, we asked if annotation-free methods also perform well. We discovered that simple and efficient models based entirely on genomic sequence content—e.g., triplets of amino acids—perform as well as intensive annotation-based classifiers, enabling rapid processing of genomes. We further show that amino acid trimers are useful because they encode information about protein composition and phylogeny. To showcase the utility of rapid prediction, we estimated the prevalence of aerobes and anaerobes in diverse natural environments cataloged in the Earth Microbiome Project. Focusing on a well-studied O_2_ gradient in the Black Sea, we found quantitative correspondence between local chemistry (O_2_:sulfide concentration ratio) and the composition of microbial communities. We, therefore, suggest that statistical methods like ours might be used to estimate, or “sense,” pivotal features of the chemical environment using DNA sequencing data.

**IMPORTANCE:**

We now have access to sequence data from a wide variety of natural environments. These data document a bewildering diversity of microbes, many known only from their genomes. Physiology—an organism’s capacity to engage metabolically with its environment—may provide a more useful lens than taxonomy for understanding microbial communities. As an example of this broader principle, we developed algorithms that accurately predict microbial dioxygen utilization directly from genome sequences without annotating genes, e.g., by considering only the amino acids in protein sequences. Annotation-free algorithms enable rapid characterization of natural samples, highlighting quantitative correspondence between sequences and local O_2_ levels in a data set from the Black Sea. This example suggests that DNA sequencing might be repurposed as a multi-pronged chemical sensor, estimating concentrations of O_2_ and other key facets of complex natural settings.

## INTRODUCTION

Dioxygen (O_2_) is a hugely consequential molecule for the biosphere. Aerobic respiration yields a tremendous amount of energy and is the most common bioenergetic mode in cells across the Earth’s surface environments. Yet O_2_ is also highly reactive, presenting challenges to organisms that encounter it (1). As a result, most genomes, whether they belong to obligate aerobes, obligate anaerobes, or facultative organisms, encode enzymes that detoxify reactive oxygen species like peroxide (H_2_O_2_) and superoxide $\left(O_{2}^{-}\right)$. The suprisingly wide distribution of detoxifying enzymes (e.g., peroxidases) and terminal oxidases (e.g., heme-copper oxidases) makes it difficult to assess which organisms are aerobic from genomes alone (2, 3).

Aerobes, however, are different from anaerobes, and these differences—though subtle—are legible in genomes. Aerobes tend to have larger genomes (4) with proteins utilizing distinct amino acids (5), a larger number of O_2_-utilizing enzymes (2), and usually belong to specific phylogenetic groups (4). Conversely, anaerobes make use of diverse fermentation pathways to conserve energy in low-O_2_ settings (3, 6). These differences have been used to predict O_2_ utilization from the genome with reasonable accuracy (2, 7, 8).

Classification of microbial O_2_ utilization typically relies on intensive preprocessing where, for example, enzymes are identified by sequence homology (2) or a full metabolic network is reconstructed (7). Such processing is costly and limited by our very incomplete knowledge of the relationship between sequence and function. We therefore asked whether accurate classification can be achieved without annotation, instead using DNA and protein sequences directly.

Classifiers trained here predict O_2_ utilization phenotypes from genomic sequences. We focused on the ternary (three-way) classification problem, categorizing organisms as (i) obligate aerobes, (ii) obligate anaerobes, or (iii) facultative. Classifiers were trained and evaluated using a compendium of ≈3,100 genomes with documented O_2_ utilization, reserving a phylogenetically balanced 20% subset for testing (Materials and Methods, Fig. S1).

## RESULTS

**Fig 1:**
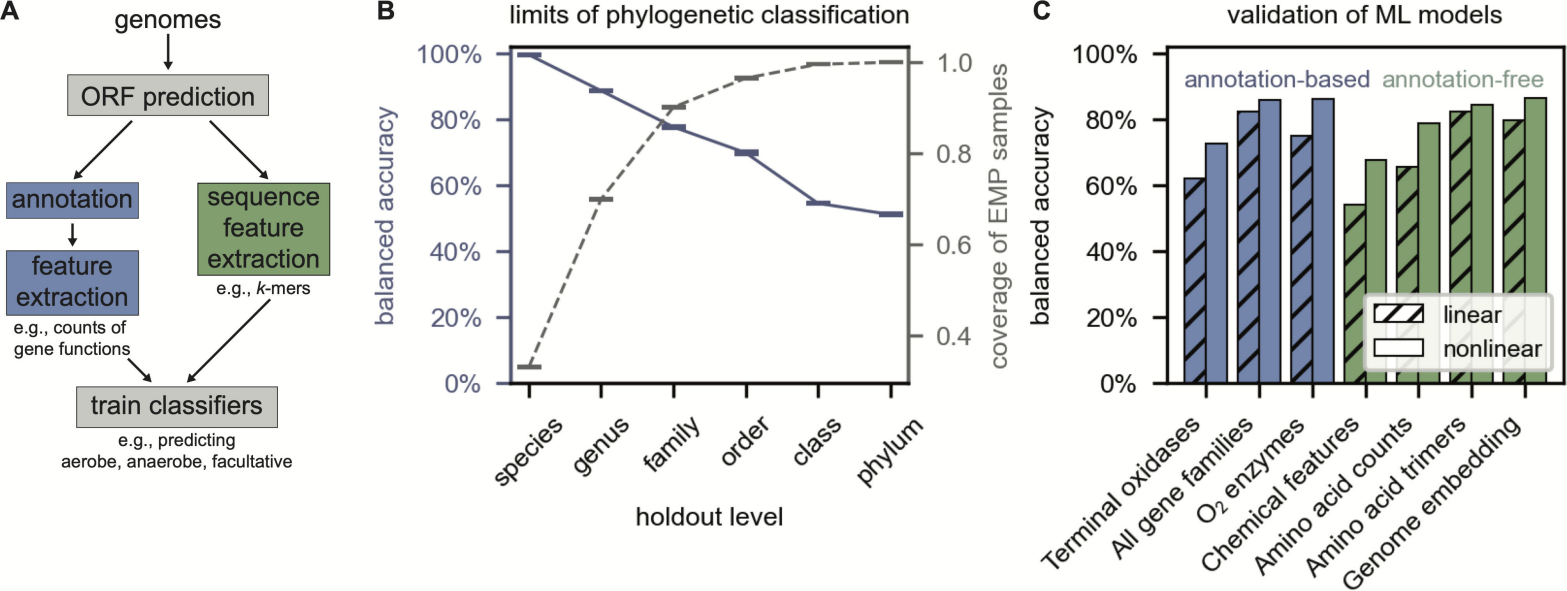
Approaches to predicting microbial O_2_ utilization. (**A**) Schematic pipelines for predicting O_2_ utilization with (left branch) and without (right) compute-intensive genome annotation. Genome annotation (≈10 min/genome) is far slower than extraction of amino acid trimers (< 1 s/genome, Table S1). (B) Close relatives are strong predictors of O_2_ utilization, as exemplified by near-perfect accuracy of “random relative” classification at the species level (solid blue curve). However, phenotypes are frequently unavailable for close relatives, as shown in gray dashes for samples from the Earth Microbiome Project (Materials and Methods) (10). (**C**) We used machine learning methods to train classifiers that can produce phenotypic predictions even for unobserved taxa. The best-performing models were ≈80% accurate at classifying out-of-sample genomes as aerobes, anaerobes, or facultative. These included annotation-free feature sets (green) like amino acid 3-mers and annotation-driven feature sets (blue) including counts of annotated protein functions (Materials and Methods). The class-balanced accuracy of guessing at random is 33% for ternary classification. Fig. S2 and Table S2 report accuracies for all models evaluated, including binary classifiers.

A typical classification pipeline begins with identification of protein-coding open reading frames (ORF prediction, Fig. 1A), followed by annotation of gene functions (2, 7, 8). Further processing is sometimes performed, e.g., constructing a metabolic network from annotations (7). Processed data are then used to train classifiers predicting phenotypes like carbon source preference (9) or O_2_ utilization from genomic features. Features can include counts of annotated gene functions, e.g., one benzoate dioxygenase, two heme oxygenases, etc. (2), or the suite of molecules produced by annotated enzymes (7, 8).

Working directly with unannotated nucleotide (NT) and amino acid (AA) sequences avoids most preprocessing steps (Fig. 1A), removes assumptions, and greatly reduces runtime (Table S1). One way of representing patterns in NT and AA sequences is by counting *k*-mers—substrings of length *k* (11, 12). A more complex, and potentially valuable, approach uses advances in machine learning to summarize (“embed”) protein sequences in vectors of fixed dimension (13, 14).

As genes and genomes are predominantly vertically inherited, any feature set, whether annotation-based or annotation-free, will be correlated with phylogeny to some degree. Indeed, related organisms have correlated O_2_ utilization (Fig. 1B). One might therefore predict the O_2_ utilization of a novel genome by querying closely related species. This approach is accurate, but its applicability is limited by the narrow taxonomic range of cultivated microbes. Generalizable prediction therefore requires a classifier integrating phylogeny with other signals.

We used linear (logistic regression) and nonlinear methods (neural networks) to train classifiers on a variety of feature sets (Materials and Methods, Fig. S2 and S3). A linear classifier predicting microbial O_2_ utilization from counts of annotated gene functions (KEGG orthogroups, Fig. 1C) displayed 82% class-balanced testing accuracy—2.5 times the accuracy of guessing at random. Yet several annotation-free classifiers also displayed ≈80% accuracy, including models based on counts of AA triplets and protein sequence embeddings. Predictions from annotation-free models also generalized well, classifying genomes from withheld phylogenetic groups substantially better than guessing at random (Fig. S4).

**Fig 2:**
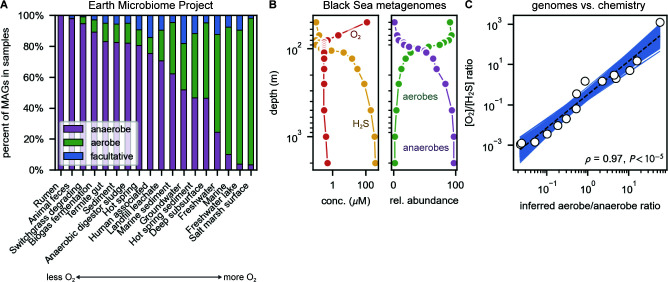
O_2_ is a major determinant of the sequence composition of natural microbial communities. (**A**) We applied our AA 3-mer classifier to metagenome-assembled genomes (MAGs) associated with Earth Microbiome Project samples (Materials and Methods). Samples from environments with characteristically low O_2_ levels (e.g., rumen and anaerobic digesters) displayed greater anaerobe content, while oxic surface environments predominantly hosted MAGs inferred to be aerobes (e.g., freshwater lakes). Fig. S7 evaluates classification using contigs instead of MAGs. (**B and C**) The Black Sea is a well-studied stratified euxinic ecosystem with a long-lived systematic O_2_ gradient—oxygenated at the surface with a loss of O_2_ and an increase in sulfide with depth. We drew depth-dependent O_2_ and H_2_S concentrations as well as Black Sea MAGs from reference 15. (**B**) We applied the nonlinear AA 3-mer model to 160 MAGs to estimate the depth-dependent prevalence of aerobes and anaerobes (Materials and Methods). Consistent with O_2_ and H_2_S profiles, aerobes were most prevalent near the surface and anaerobes most prevalent at depth. (**C**) The [O_2_]/[H_2_S] ratio was strongly correlated with the inferred aerobe/anaerobe ratio on a log–log plot (Pearson $\rho =0.97,P<10^{-5}$) such that estimating the redox gradient from sequencing data resulted in <80% relative error over roughly six orders.

Counting AA triplets is far more efficient than annotating genomes, which greatly accelerated our evaluation of O_2_ utilization in environmental samples. To demonstrate the utility of rapid characterization, we analyzed ≈30,000 metagenome-assembled genomes (MAGs) from Earth Microbiome Project samples (10) using the nonlinear AA 3-mer model. Consistent with expectations, samples of characteristically anaerobic habitats (e.g., rumen, 606/606 predicted anaerobic MAGs) contained a much greater proportion of anaerobic MAGs (Fig. 2A).

To examine the quantitative relationship between local chemistry ([O_2_]) and physiology (O_2_ utilization), we applied the AA 3-mer model along a natural O_2_ gradient (Fig. 2B). Due to its unique hydrography and intense density gradient, the surface of the Black Sea mixes poorly with deeper waters, leading to a sharp transition from oxia near the surface to anoxic and sulfidic habitats at depth (15, 16). As expected from these chemical transitions, the classifier predicted sympathetic traces of O_2_ utilization with depth, with aerobic MAGs dominating near the surface and anaerobes in deeper waters below the mixed layer (Fig. 2C). This correspondence was also quantitative, with the [O_2_]/[H_2_S] ratio correlating strongly with inferred aerobe/anaerobe ratios, suggesting that chemical gradients might be “sensed” by analysis of DNA sequences (Fig. 2C).

## DISCUSSION

In this study, we evaluated classifiers of microbial O_2_ utilization phenotypes. While typical approaches rely on annotated genomes (2, 7, 8), we found that several computationally efficient annotation-free models performed similarly to the best annotation-driven approaches (Fig. 1C).

There are two compatible explanations for the success of these naive models. First, O_2_ utilization is correlated with phylogeny to a degree (4), and *k*-mer counts are a proxy for phylogenetic proximity (17–19). A phylogenetic cross-validation (Fig. S4) revealed that (i) related species indeed have correlated O_2_ utilization and (ii) classifiers rely on phylogenetic correlations to varying degrees. Predictions based on phylogeny can be useful when the species of interest have relatives in the training set (e.g., Fig. 1B). Illustrating the potential of phylogenetic predictions, we achieved useful prediction accuracies with a classifier trained on machine-learned embeddings of ribosomal 16S sequences (Fig. S5).

A second explanation for the success of *k*-mer models posits that the contents of genomes and proteomes adapts to the reactivity of O_2_, e.g., by incorporating fewer redox-active groups like cysteine thiols (5). To evaluate this argument, we trained classifiers on (i) genomic AA counts (1-mers) and (ii) chemical descriptors of AAs and NTs (e.g., elemental content and C redox state, see Materials and Methods). Such features contain less phylogenetic information yet produce classifiers that perform much better than guessing at random (Fig. 1C; Fig. S2). As such, a practical advantage of *k*-mers is that they encode chemical and phylogenetic information, indicating that *k*-mers may simplify prediction of other complex phenotypes (9).

Our exploration of metagenomes indicated that the physical and chemical conditions of natural environments affect their sequence content in a legible way (Fig. 2). Indeed, we observed a quantitative correspondence between local chemistry ([O_2_]/[H_2_S]) and inferred aerobe/anaerobe ratios in the Black Sea (Fig. 2C), suggesting that the inverse problem—estimating the concentrations of O_2_ and other key molecules from sequence data—is tractable. Microbes use a wide variety of genetically encoded mechanisms to extract and utilize species of phosphorus, nitrogen, sulfur, and carbon. Similar to O_2_, we expect the presence and abundance of microbial taxa extracting mineral phosphorus to relate to soluble phosphorus concentrations, for example.

Nutrient supply (e.g., N, P, and Fe) limits the growth of crops and the productivity of ecosystems, yet it is currently very challenging to characterize or monitor environmental chemistry at a frequency or scale useful for agriculture or Earth system models. Our results here suggest that sequencing data could serve as a “multi-sensor” of the local, biologically available concentrations of key nutrients. Substantial research is needed to realize this vision, collecting and collating environmental sequencing data with paired chemical measurements, potentially learning the genetic mechanisms by which microbial taxa access nutrients, and, finally, calibrating models inferring nutrient concentrations (or fluxes) from such data. Yet, if such efforts enable scalable monitoring of diverse microbial habitats, they are surely worthwhile.

## MATERIALS AND METHODS

### Training, validation, and testing data sets

The data sets of Madin et al. (20) and Jabłońska & Tawfik (2) were merged and mapped onto the Genome Taxonomy Database (GTDB release r207 [21]) to produce a collection of genomes and metagenomes with known modes of dioxygen utilization. Reference 2 provides RefSeq IDs, which were used to retrieve genomes and coding sequences. Roughly 350 genomes not meeting NCBI quality standards (marked “suppressed”) were then removed. We generated three classes of labels for each genome using the following rules: we labeled annotations “Anaerobe” and “Obligate anaerobe” as “Anaerobe,” “Facultative” and “Facultative anaerobe” as ”Facultative,” and “Aerobe,” “Microaerophilic,” and “Obligate aerobe” as ”Aerobe.” See Fig. S8 for the distribution of raw labels. Genomes were processed with a custom Python pipeline to extract features (e.g., nucleotide tetramers). Genome annotation was performed using kofamscan (22), and protein embedding was performed with the protein language model ProtT5-XL-uniref50 (13). The merged data set was then split by reserving 20% of genomes in each phylogenetic class for an independent test set. Twenty percent of the remaining genomes were reserved for a validation set used in hyperparameter selection for nonlinear models. These withheld sets are phylogenetically representative of the training set (Fig. S1), so validation and testing represent phylogenetic “interpolation tests”—i.e., test the model’s ability to predict phenotypes of microbes related to those in the training set at the class level or closer.

### Feature sets tested

We developed a common pipeline to evaluate 21 feature sets (Fig. S2). Annotation-free feature sets included the number of predicted open reading frames (“gene count”), counts of genomic DNA *k*-mers (lengths 1–5), counts of coding sequence (CDS) nucleotide *k*-mers (lengths 1–5), CDS amino acid *k*-mers (lengths 1–3), a list of simple chemical features of nucleotide and amino acid sequences in each genome (“chemical features”), and genome embeddings. Chemical features included the number of open reading frames, genomic GC content, the average number of carbon, nitrogen, oxygen and sulfur atoms (23) per monomer (AA or NT) in protein- and RNA-coding sequences, as well as the average redox state of carbon ($Z_{C}$) in those same sequences (5). Genome embeddings were generated by first passing all protein-coding sequences through a pretrained large language model (13) and mean-pooling each protein embedding over sequence length to produce one fixed-length 1,024-dimensional vector per sequence. Then, for each genome, we averaged the protein embeddings to produce a “genome embedding.” Annotation-based feature sets included per-genome counts of KEGG orthogroups (“All gene families”), per-genome counts of terminal oxidases, mean embeddings of all annotated O_2_-utilizing enzymes in each genome (“O_2_ enzymes”), and two scalar feature sets: the number of O_2_-utilizing enzymes and the fraction of genes that are O_2_-utilizing enzymes.

### Model training

We applied both linear and nonlinear classifiers to estimate the mapping between features and labels. Our linear method was $L_{2}$-regularized logistic regression (Python sklearn package; regularization strength set to C = 100, max_iter = 10,000), which we used to compare binary (O_2_-tolerant vs O_2_-intolerant) and ternary classifiers (aerobe vs anaerobe vs facultative) of O_2_ utilization. We also evaluated a neural network by using a candidate nonlinear method. Using the PyTorch package, we implemented simple $L_{2}$-regularized multilayer perceptron consisting of an input layer, a 512-node hidden layer, and a three-dimensional output layer. For all feature sets, the nonlinear model was trained on batches of size 16 for at most 100 epochs, with a learning rate of 0.0001. Final model weights were selected based on accuracy on the validation set. Throughout, we report class-balanced accuracies calculated using the sklearn.metrics package in Python. Model accuracies are summarized in Fig. S2 and Table S2 with per-class accuracies reported in Fig. S3 for select models.

### Phylogenetic cross-validation

To test whether models generalize well to genomes from withheld phylogenetic groups, we designed a phylogenetic cross-validation scheme. We used sklearn’s GroupShuffleSplit to generate five random splits each withholding ≈20% of genomes from the training set. This method ensures that all members of a particular phylogenetic group—e.g., family or class—are either in the training set or withheld. At the class level, for example, this entails withholding all of the Chlorobia or none of them. We then trained each model on the remainder of the training set and evaluated the accuracy on withheld genomes to produce balanced accuracies as a function of phylogenetic holdout level for each model, as shown in Fig. S4. As a baseline, we implemented a “random relative“ classifier that is based solely on phylogeny. To predict O_2_ utilization, the random relative classifier chooses a genome that belongs to the same phylogenetic group at the prescribed level. For a query genome in the class Chlorobia, for example, another genome in the same class would be selected at random. This phylogenetic approach is very accurate when phenotypic information is available for closely related species, but fails to produce predictions at all when this is not the case (Fig. S4).

### Classification using embeddings of 16S sequences

To predict O_2_ utilization from 16S rRNA DNA sequencing, we applied the pretrained DNA Language Model GenSLM (24). For genomes with NCBI accession for 16S rRNA genes, we extracted the V34 region and embedded this into a 512-dimensional vector using GenSLM. NCBI accessions were not available for all genomes in (20), meaning that 16S sequences could not be ascertained in all cases. This resulted in a data set of n=1,031 variable regions from genomes with known oxygen requirements. We randomly partitioned the data into training sequences (n=693), validation sequences (n=150), and testing sequences (n=188). We constructed a classification layer on top of GenSLM in PyTorch, varying only the weights in this additional layer during training. The model was trained with a learning rate of 0.01, a batch size of 16, and for a maximum of 100 epochs using the Adam optimizer. The final model was chosen via early stopping at epoch 95, which corresponded to the model with the highest balanced accuracy for the validation set during training. Note that this model uses different training and test sets than models trained on full genomes. As such, these model results are not directly comparable with those in Fig. 1 ; Fig.S2 , so they are presented separately in Fig. S5.

### Black Sea analysis

Paired chemical measurements and DNA sequencing data were drawn from (15), which assembled metagenome-assembled genomes (MAGs) from Black Sea samples. The relative abundances of MAGs were estimated previously in (25). Briefly, metagenomic samples were aligned to the previously-assembled MAGs using bbmap. Alignments with a mapping quality above 10 were retained, converted to BAM format, sorted, and indexed using samtools. The relative abundance of each MAG was determined by the fraction of reads mapped to it, as summarized by samtools idxstats. This process was automated via a Python script, utilizing samtools v1.8 and bbmap.sh, and executed on the Resnick High-Performance Computing Center cluster at Caltech. As we achieved competitive accuracy using a nonlinear classifier trained on amino acid trimers, we applied this model to the Black Sea MAGs.

### Earth Microbiome Project (EMP) analysis

O_2_ utilization phenotypes of EMP metagenome-assembled genomes from (10) were classified using the nonlinear AA 3-mer model. As EMP projects are categorized with an *ad hoc* nomenclature describing the environment sampled, we manually mapped tags to a simplified set of categories. For analysis, we removed MAGs with less than 50% estimated completeness, considered only samples from which at least 10 MAGs were assembled and only environmental labels (e.g., “rhizosphere”) for which at least 10 samples were available (see Fig. S6). This left 1,598 samples and 31,279 MAGs for consideration. The data presented in Fig. 2C give the fraction of MAGs that are inferred to be aerobes, anaerobes, and facultative in each habitat for samples meeting these criteria.

### Contigs as predictors of dioxygen utilization

We used MAGs in our above-described analysis of environmental samples. As MAG binning is compute-intensive and model-dependent, it may be preferable to evaluate O_2_ utilzation from contigs or raw reads directly. To determine if such an analysis is feasible, we evaluated model performance on artificial contigs generated from 100 genomes in the testing set. For a genome of size n and contigs of length l, n/l non-overlapping contigs were generated and written to a FASTA file. Predictions were generated by running nonlinear nucleotide models—NT 3-, 4-, and 5-mer—on per-contig feature vectors. Using NT features avoids the use of open reading frame prediction, which is more complex for partial sequences like contigs. As shown in Fig. S7, relatively long contigs (>10 kbp) were required for local predictions to match the global one.

## Supplementary Material

Reviewer comments

## Data Availability

Source code is available at github.com/flamholz/annotation_free_dioxygen_utilization. A provided script automates retrieval of data from the figshare repository at https://figshare.com/articles/dataset/Annotation-free_prediction_of_microbial_dioxygen_utilization/26065345.

## SUPPLEMENTAL MATERIAL

Supplemental material msystems.00763-24-s0001.pdf Supplemental figures and tables.

OPEN PEER REVIEW reviewer-comments.pdf An accounting of the reviewer comments and feedback.

ASM does not own the copyrights to Supplemental Material that may be linked to, or accessed through, an article. The authors have granted ASM a non-exclusive, world-wide license to publish the Supplemental Material files. Please contact the corresponding author directly for reuse.
