# Supplementary material for: Annotation-free prediction of microbial dioxygen utilization
Source: mSystems. 2024 Sep 4;9(10):e00763-24. doi: 10.1128/msystems.00763-24 (PMC11494890; doi:10.1128/msystems.00763-24)
Supplement: Supplemental material — Supplemental figures and tables. [file msystems.00763-24-s0001.pdf]

SUPPLEMENTARY FIGURES & TABLES

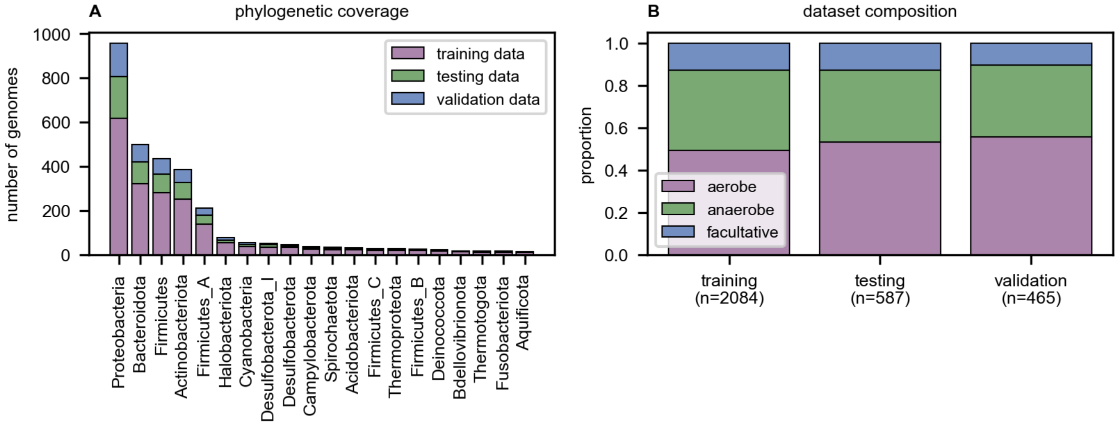

FIG S1 Summary of the training, validation and test sets. Panel (A) displays counts from the 20 phyla with the most representatives. Panel (B) gives the composition of training, test, and validation sets in terms of O_2 utilization.

TABLE S1 The running time of of genome annotation far exceeds that of *k*-mer extraction. Mean and standard deviations of running times on an Apple laptop computer.

	mean runtime (s)	standard deviation
feature_type		
3mer	0.35	0.13
annotation	1326.84	49.25

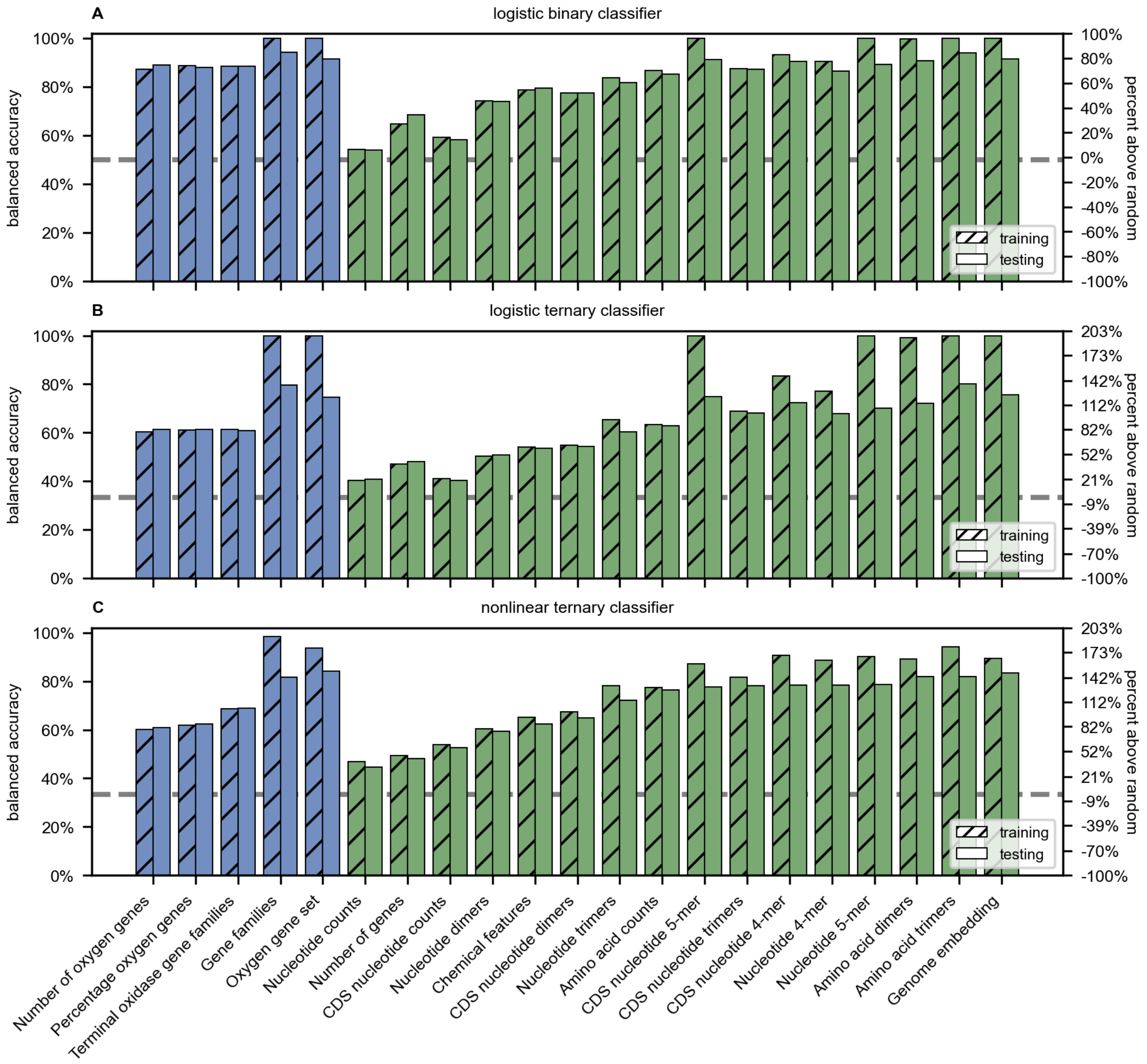

FIG S2 Training and test-balanced accuracies for all models. Models are either annotation-full (blue, left) or annotation-free (green, right). Within groups, models are ordered by increasing balanced accuracy on the test set when using a non-linear ternary classifier. Hatched bars represent training accuracy, while testing accuracy are given as solid bars. Panel (A) shows the results of logistic regression for the binary classification problem, classifying genomes as O_2 tolerant or intolerant, (B) logistic regression for ternary classification, and (C) a nonlinear classifier ternary classifier (Methods). Dashed gray lines mark the random guessing threshold – 33% balanced accuracy for ternary classifiers and 50% accuracy for binary. See Methods for detailed description of individual models.

TABLE S2 Summary of balanced accuracy for each classifier evaluated here. Fractional testing accuracies are given in descending order by performance of the nonlinear ternary classifier (training accuracy in parentheses). Note that three of the top five non-linear models relied on annotation-free feature sets: genome embeddings, amino acid dimers, and amino acid trimers.

feature type	nonlinear ternary	logistic ternary	logistic binary
Oxygen gene set	0.84 (0.94)	0.75 (1.00)	0.92 (1.00)
Genome embedding	0.83 (0.89)	0.76 (1.00)	0.91 (1.00)
Amino acid trimers	0.82 (0.94)	0.80 (1.00)	0.94 (1.00)
Amino acid dimers	0.82 (0.89)	0.72 (0.99)	0.91 (1.00)
Gene families	0.82 (0.99)	0.80 (1.00)	0.94 (1.00)
Nucleotide 5-mer	0.79 (0.90)	0.70 (1.00)	0.89 (1.00)
Nucleotide 4-mer	0.78 (0.89)	0.68 (0.77)	0.87 (0.91)
CDS nucleotide 4-mer	0.78 (0.91)	0.72 (0.83)	0.91 (0.93)
CDS nucleotide trimers	0.78 (0.82)	0.68 (0.69)	0.87 (0.88)
CDS nucleotide 5-mer	0.78 (0.87)	0.75 (1.00)	0.91 (1.00)
Amino acid counts	0.76 (0.77)	0.63 (0.63)	0.85 (0.87)
Nucleotide trimers	0.72 (0.78)	0.60 (0.65)	0.82 (0.84)
Terminal oxidase gene families	0.69 (0.69)	0.61 (0.61)	0.89 (0.89)
CDS nucleotide dimers	0.65 (0.67)	0.54 (0.55)	0.78 (0.77)
Chemical features	0.62 (0.65)	0.54 (0.54)	0.79 (0.79)
Percentage oxygen genes	0.62 (0.62)	0.61 (0.61)	0.88 (0.89)
Number of oxygen genes	0.61 (0.60)	0.61 (0.60)	0.89 (0.87)
Nucleotide dimers	0.59 (0.60)	0.51 (0.50)	0.74 (0.74)
CDS nucleotide counts	0.53 (0.54)	0.40 (0.41)	0.58 (0.59)
Number of genes	0.48 (0.49)	0.48 (0.47)	0.69 (0.65)
Nucleotide counts	0.45 (0.47)	0.41 (0.40)	0.54 (0.54)

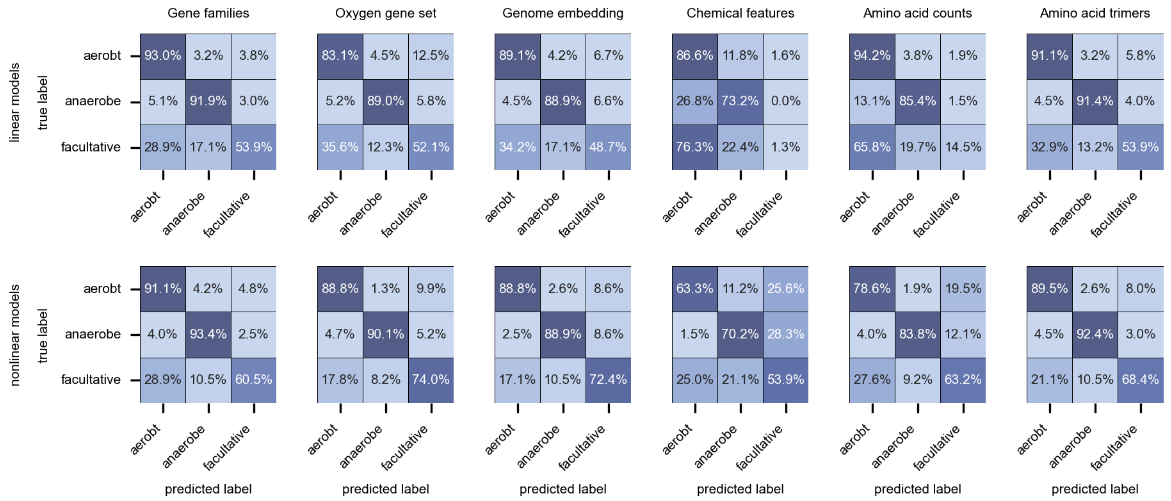

FIG S3 Confusion matrices for a selection of ternary classifiers. Linear models (logistic regression) are in the top row while nonlinear model performance is in the second row. Confusion matrices depict per-class testing accuracies. See Methods for detailed description of feature sets and model implementation. Notice that, in all cases, prediction accuracy was lowest for facultative organisms.

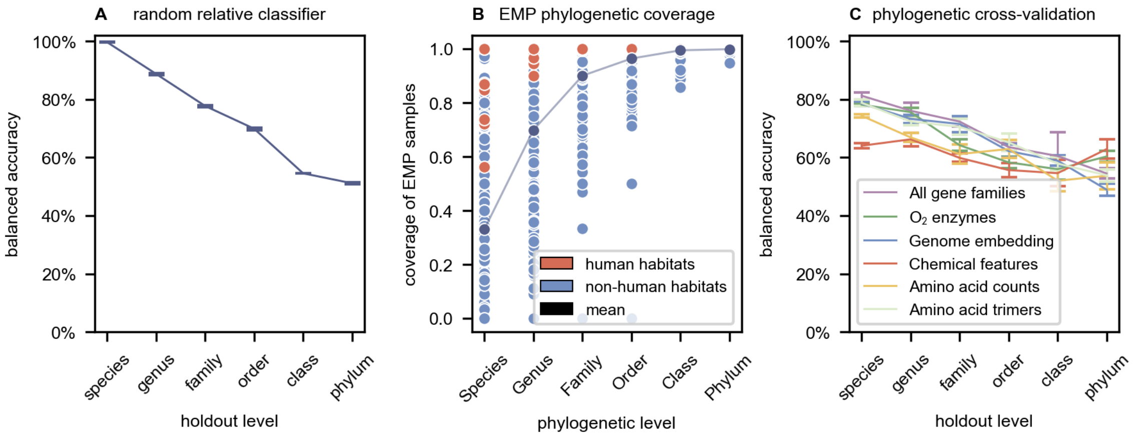

FIG S4 Phylogeny is a useful, but limited, predictor of O_2 utilization. (A) The 'random relative' classifier assigns a O_2 utilization tag to a novel genome by choosing a random relative at a prescribed phylogenetic level (e.g. species or genus, see Methods). This approach is accurate, especially when data on close relatives is available. Yet, as panel (B) shows, such data are often unavailable. Considering samples from the Earth Microbiome Project [9], we found that reconstructed MAGs often belong to species and genera for which phenotypes are not available (i.e. low coverage). Statistical models can use phylogenetic information along with other signals to classify genomes that any genome – i.e. such classifiers have 100% coverage. We evaluated the degree to which such models rely on phylogenetic signal by performing a cross-validation wherein whole groups were omitted from the training set (Methods). Results are plotted in panel (C) for nonlinear ternary classifiers. Classifier accuracy degrades when relatives are omitted from the training set, and this effect is more pronounced (lower accuracy on holdout) when larger groups of organisms are omitted (e.g. orders instead of genera). Nonetheless, prediction accuracy remained well in excess of the 33% accuracy of blind guessing.

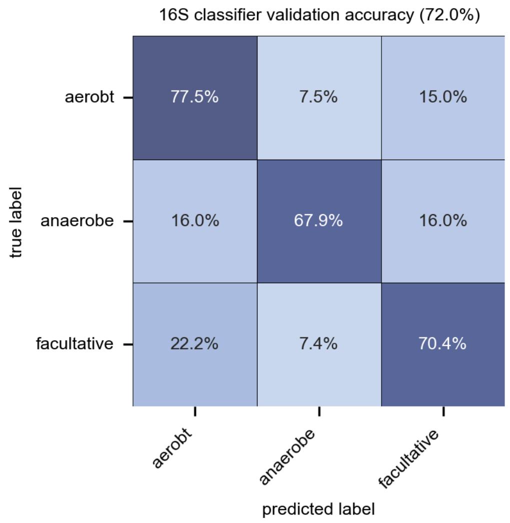

FIG S5 A predictor based on embedding 16S sequences performs similarly to other approaches, but with a distinct error profile. We used machine-learning driven DNA sequence embedding to develop a classifier based on 16S rRNA sequences. Due limitations of the source data, balanced accuracy is here calculated over a smaller, randomized test set (Methods). Accuracy values are therefore not directly comparable to Figs. **1**, S2 or S3.

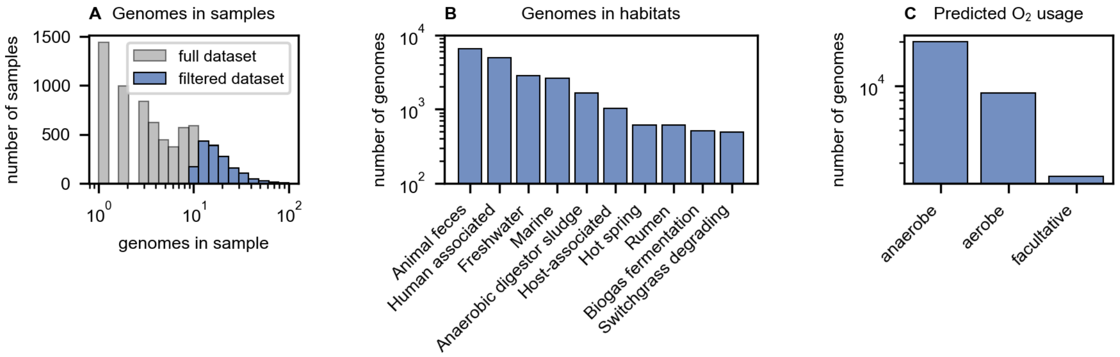

FIG S6 Summary of the Earth Microbiome Project (EMP) metagenomic compendium. (A) The number of metagenome-assembled genomes (MAGs) associated with individual samples was non-uniform. We filtered the dataset to retain higher-quality MAGs and samples (Methods). (B) Sample habitats were broadly categorized so that samples could be grouped and compared by environment. Here we show habitats with the largest number of associated samples. (C) Counts of O_2 utilization as inferred by running the nonlinear AA 3-mer model on EMP MAGs.

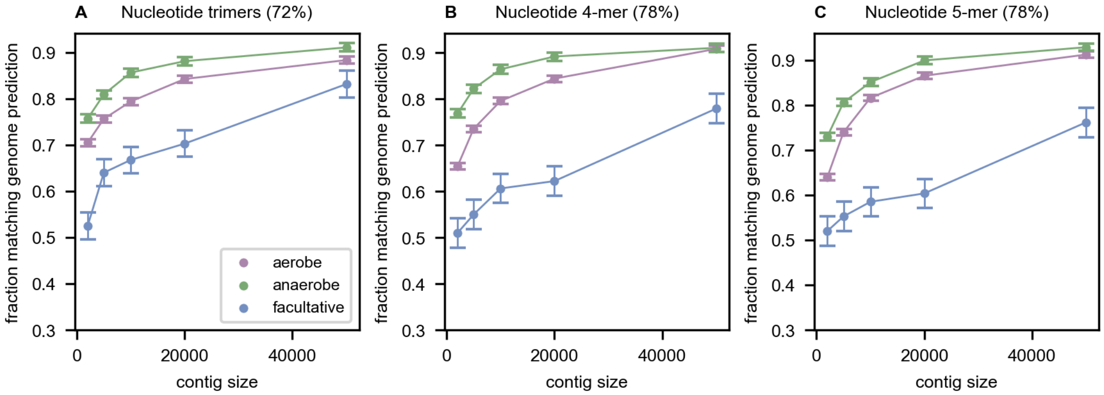

FIG S7 Evaluation of contigs as predictors of microbial O_2 utilization. We generated all possible contigs of a given length l from validation set genomes, which have known O_2 utilization (Methods). Relatively long contigs were required to match classifications made using the whole genome. Panel (A) shows results using the non-linear nucleotide (NT) trimer model, (B) NT 4-mer and (C) NT 5-mers. As discussed in the Methods section, we chose to apply the NT models here to avoid the complexity of open reading frame prediction from partial sequences.

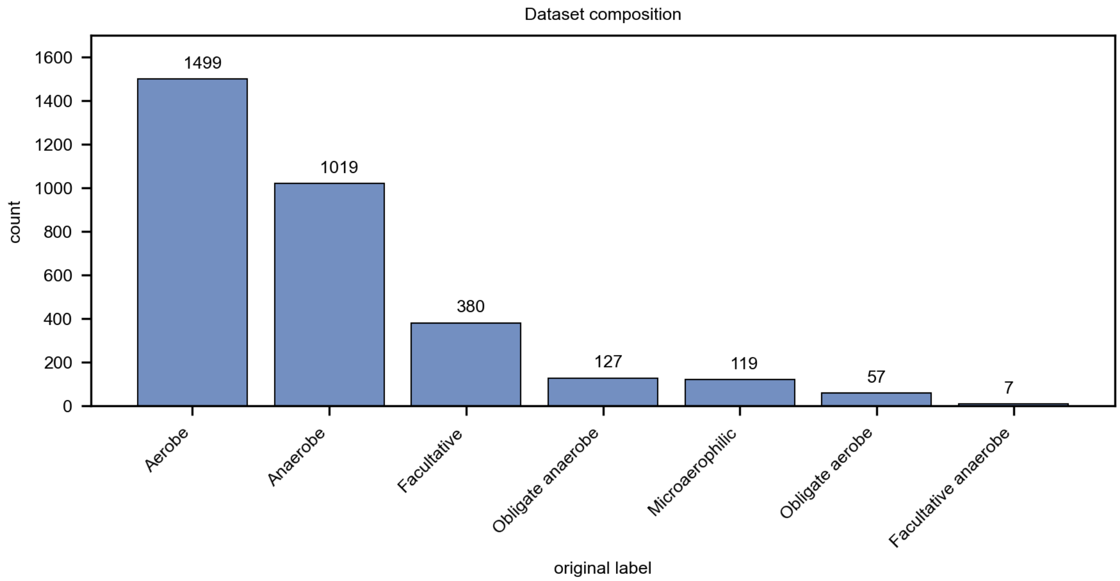

FIG S8 Distribution of raw O_2 utilization tags in [20].
